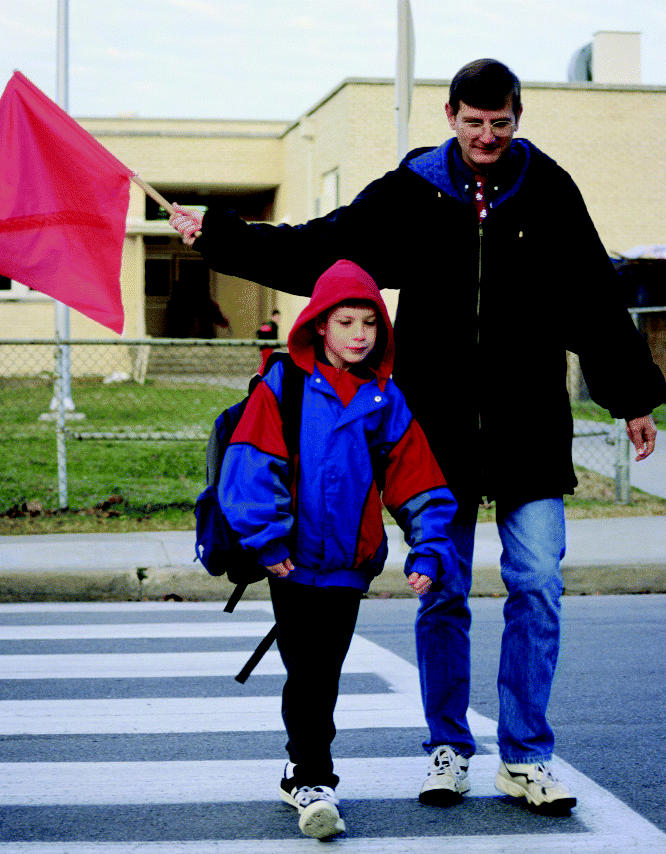# Symposium Explores Children’s Environments

**DOI:** 10.1289/ehp.112-a990

**Published:** 2004-12

**Authors:** Martha M. Dimes

“When we try to pick out anything by itself, we find it hitched to everything else in the Universe.” Quoting naturalist John Muir, Michael Fischer, an environmental consultant formerly of the William and Flora Hewlett Foundation, introduced the 2004 Biennial Scientific Symposium on Children’s Health as Impacted by Environmental Contaminants by emphasizing that children are at the nexus of many of the connections found in nature.

The symposium was designed to explore the interconnectedness of all elements of the environments in which children live, learn, and play, as well as ways to prevent environmental health risks. Hosted by the Children’s Environmental Health Institute (CEHI), the symposium was held 24–25 September 2004 at the McKinney Roughs Nature Park in Austin, Texas. The symposium was sponsored by the NIEHS, Physicians for Social Responsibility, the Texas Medical Association, the Lower Colorado River Authority, and the Centers for Disease Control and Prevention. The interdisciplinary group of participants included researchers, pediatricians and other health professionals, social workers, nonprofit and advocacy group representatives, architects, and engineers.

Topics ranged from the cellular level to the global. Because environmental toxicants are ubiquitous in air, water, food, and medications, said pediatrician and speaker Martin Lorin, we have seen a global rise in environmentally related diseases. However, in terms of the extent of the effects of exposures, he contended, we are seeing only the tip of the iceberg.

Participants agreed that to reduce harmful immediate and long-term effects of contaminants on children, we must study the interactions of environment, genes, developmental stage, and behavior. Discussions on endocrine disruptors (by John McLachlan of Tulane and Xavier Universities), developmental defects (by Richard Finnell of Texas A&M University), and respiratory disease (by Sharon Petronella of The University of Texas Medical Branch, Galveston), for example, addressed not just specific immediate health problems in children but also future trends: What kinds of adult diseases might be projected from fetal and childhood exposures? And how healthy are future populations likely to be?

Potential remedies for environmentally related health problems may be as simple as taking folic acid to help prevent birth defects or having a physician take an environmental history to spot potential health risks. Technical (air quality samplers, hand-held immunosensors, microarrays) and demographic (geographic information systems, longitudinal studies) tools also can help identify and alleviate environmental health threats.

The built environment—both materials and design—can significantly reduce children’s exposures to toxicants while creating safe and stimulating places to grow and learn. How do we replace the persistent bioaccumulative and toxic chemicals used to produce building materials with less-toxic alternatives?

Gail Vittori, codirector of the Center for Maximum Potential Building Systems, suggested several methods, among them eliminating interior finish materials that offgas volatile organic compounds, using recycled fly ash as a substitute for concrete, labeling building products more thoroughly, and using paints certified by the independent Green Seal standards program.

Vittori also recommended that builders participate in the Leadership in Energy and Environmental Design program, a voluntary standard established by the U.S. Green Building Council for assessing and certifying high-performance sustainable buildings. In schools, inadequate ventilation, use of toxic pesticides and cleaners, offgassing from building materials and furnishings, and poor maintenance should be remediated to avoid increases in asthma, allergies, and other respiratory diseases.

With an eye toward the future, the NIEHS and the U.S. Environmental Protection Agency will continue to fund the Centers for Children’s Environmental Health and Disease Prevention Research, according to NIEHS director Kenneth Olden. These centers promote multidisciplinary research and the translation and application of research to public health and clinical practice. The Centers for Disease Control and Prevention aims to expand environmental public health tracking to a full nationwide network, collecting and analyzing data on hazards, exposures, and health effects. And Fernando Guerra, director of health with the San Antonio Metropolitan Health District, noted that the multi-agency National Children’s Study “will provide for the first time an opportunity for children and families to benefit from the cumulative evidence that will be assembled over twenty-five years, to better understand causal relationships from many different influences, including the environment.”

The participants concluded that prevention, remediation, and attention to the long term are essential to addressing the unique vulnerabilities of infants and children. The challenge presented here is to blend research and clinical work with advocacy. Said CEHI director Janie Fields: “Together we are building a structure that bridges the health information gap between the medical, research, and environmental communities.”

## Figures and Tables

**Figure:**